# Information collected during the post-breeding season guides future breeding decisions in a migratory bird

**DOI:** 10.1007/s00442-020-04629-5

**Published:** 2020-03-12

**Authors:** Jere Tolvanen, Chiara Morosinotto, Jukka T. Forsman, Robert L. Thomson

**Affiliations:** 1 Department of Ecology and Genetics, University of Oulu, 90014 Oulu, Finland; 2 National Resources Institute Finland, University of Oulu, 90014 Oulu, Finland; 3 Section of Ecology, Department of Biology, University of Turku, 20014 Turku, Finland; 4 Bioeconomy Research Team, Novia University of Applied Sciences, Raseborgsvägen 9, 10600 Ekenäs, Finland; 5 FitzPatrick Institute of African Ornithology, DST-NRF Centre of Excellence, University of Cape Town, Rondebosch, 7701 South Africa

**Keywords:** Habitat choice, Reproductive investment, Social information, Between-individual variation, *Ficedula*

## Abstract

Breeding habitat choice and investment decisions are key contributors to fitness in animals. Density of individuals is a well-known cue of habitat quality used for future breeding decisions, but accuracy of density cues decreases as individuals disperse from breeding sites. Used nests remain an available information source also after breeding season, but whether such information is used for breeding decisions is less well known. We experimentally investigated whether migratory, cavity-nesting pied flycatchers (*Ficedula hypoleuca*) prospect potential breeding sites after breeding season and use old nests as a cue for future breeding decisions. In late summer 2013, forest sites were assigned to four treatments: (1) sites including nest boxes with old nests of heterospecifics (tits), (2) sites including suitable but empty nest boxes, (3) sites with unsuitable nest boxes, or (4) sites without any nest boxes. In the following year, we investigated pied flycatcher habitat choice and reproductive investment according to these “past” cues while also controlling for additional information sources present during settlement. Flycatchers preferred sites where tits had been perceived to breed in the previous year, but only if great tits were also currently breeding in the site and had a relatively high number of eggs. Old flycatchers avoided sites previously treated with suitable but empty cavities, whereas young flycatchers preferred sites where tits had apparently bred in the previous year. Also egg mass, but not clutch size or clutch mass, was affected by the combination of past treatment information and current tit abundance.

**Electronic supplementary material:**

The online version of this article (10.1007/s00442-020-04629-5) contains supplementary material, which is available to authorized users.

## Introduction

Breeding habitat choice is an important decision for animals by defining available resources and risks for both breeding adults and their progeny. Thereby, breeding habitat choice affects individual fitness during the breeding season and also in future life (Pärt 2001; Sergio et al. 2009). Animals may improve habitat choice and investment decisions by considering information on relative habitat quality by personally interacting with environment and using social information, based on both conspecific and heterospecific individuals (Danchin et al. 2004; Seppänen et al. 2007; Goodale et al. 2010; Schmidt et al. 2010). Especially in birds, social information use is widespread in breeding habitat choice (Szymkowiak 2013). Both colonial and solitarily breeding birds use conspecific density and distribution (Doligez et al. 2004b; Sebastián-Gonzáles et al. 2010; Kivelä et al. 2014), breeding success (Doligez et al. 2002; Sergio and Penteriani 2005; Ward 2005; Parejo et al. 2007a; Boulinier et al. 2008; Kelly and Schmidt 2017), and individual quality (Szymkowiak et al. 2016) to adjust breeding site choices.

Conspecifics are valuable information sources, but availability of within-season conspecific information may be limited, at least for the earliest settling part of a population. Individuals of other species often comprise the majority of individuals in the landscape, and thus, availability of information may be greatly increased if also heterospecifics are included as information sources. Earlier breeding heterospecifics also may reveal information that is not yet available from conspecifics (e.g., of breeding success). Accordingly, a variety of migratory birds use heterospecific information for habitat choice and investment decisions within a breeding season (Forsman et al. 2002; Thomson et al. 2003; Hromada et al. 2008; Sebastián-Gonzáles et al. 2010; Parejo et al. 2012; Loukola et al. 2013; Kivelä et al. 2014; Szymkowiak et al. 2017; Tolvanen et al. 2018). Whether heterospecific cues are assessed during or after a breeding season to aid habitat choice in future breeding attempts is, however, less well known (Parejo et al. 2005; Sebastián-Gonzáles et al. 2010; Forsman et al. 2014; Kivelä et al. 2014).

Birds acquire social information through actively prospecting the behavior, nests, and fledglings of other individuals (Reed et al. 1999; Schjørring et al. 1999; Doligez et al. 2004a; Forsman and Thomson 2008; Parejo et al. 2008; Thomson et al. 2013). This behavior is frequent during the nestling period when value of the extracted information is highest (Schjørring et al. 1999; Doligez et al. 2004a). Prospecting during breeding may, however, be constrained by activities necessary to take care of offspring (Reed et al. 1999; Schjørring et al. 1999; Doligez et al. 2004a; but see Thomson et al. 2013; Moks et al. 2016). In parallel, nestlings are incapable of prospecting other nests (at least in solitarily breeding birds, e.g., Doligez et al. 2004a; Parejo et al. 2007b), but after fledging of the young and especially after the fledgling-dependence period, both breeding adults and fledglings may allocate more time in prospecting (Ward 2005; Arlt and Pärt 2008). Fledglings and immatures show considerable movement in the post-breeding period before migration, a potential sign of prospecting for future breeding sites (Péron and Grémillet 2013; Brown and Taylor 2015; Vega et al. 2016; Krüger and Amar 2017). On the other hand, breeding adults and their offspring often remain close (< 100 m) to their nest sites for less than 10 days after fledging (Streby and Andersen 2013; van Overveld et al. 2017; Kysučan et al. 2020). Therefore, the post-breeding social cues based on the density of adults and fledglings, or their proxies, such as fledgling call rates (Nocera et al. 2006; Betts et al. 2008; Kelly and Schmidt 2017) may be spatially accurate for only relatively short period of time. Another potential social cue that may be used for assessing habitat quality, even after breeding birds and fledglings have left an area, is presence and contents of old nest structures.

Nest-site choice in relation to old conspecific nests present in the beginning of breeding season has been studied before (reviewed in Mazgajski 2007). However, the information value of old nests has rarely been distinguished from other hypotheses (e.g., parasite infection risk and nest construction benefits; Mazgajski 2007), and the few exceptions provide contrasting results (Erckmann et al. 1990; Yahner 1993; Gergely et al. 2009). Whether old nests of other species, observed during the post-breeding period, could be used as a valuable information source to select high-quality breeding sites in the next breeding season remains unknown. An ability to use old nests or nest contents, including those of other species, as social information about habitat quality would considerably increase available habitat quality information. Besides these social cues, mere presence or abundance of available nest sites (e.g., cavities) could be used as a cue for future habitat choice. Secondary cavity-nesting birds could also obtain information on the new cavities made by woodpeckers or other cavity excavators during the breeding season.

We conducted a field experiment to test whether a migratory cavity-nesting passerine, the pied flycatcher *Ficedula hypoleuca*, uses heterospecific social information, expressed by nest remains of the great tit *Parus major*, or information on the abundance of suitable nest sites (empty cavities), to adjust its breeding site choice and investment decisions during the next breeding season. We isolated the availability of this information only to the post-breeding period (from fledging of the young until autumn migration). Pied flycatcher is an ideal model for investigating these questions, because it readily incorporates social information in settlement and investment decisions (e.g., Forsman et al. 2002, 2012; Loukola et al. 2013; Samplonius and Both 2017; Tolvanen et al. 2018).

Diverse use of various social information sources during breeding season (short-term use) may obscure long-term (between-years) effects of a specific cue. Therefore, we investigated the effects of the long-term experimental cues while controlling for short-term social information sources present during settlement, i.e., the abundance and success of conspecifics and heterospecific tits (e.g., Forsman et al. 2002, 2012; Loukola et al. 2013; Kivelä et al. 2014; Samplonius and Both 2017; Tolvanen et al. 2018). If flycatchers collect information about the quality or abundance of potential future breeding sites during the post-breeding period, and if they are capable of cueing on mere nest cavity contents, we expect them to preferentially settle in sites where suitable nest sites were abundant. In addition, we expect that birds will especially prefer and show higher reproductive investment in sites where heterospecific tits were apparently breeding in the previous year.

## Materials and methods

### Experimental design

We conducted the experiment in 2013–2014 and replicated it in two study areas, ca. 250 km apart, in western Finland, close to the cities of Oulu (65° N 26° E) and Kauhava (63° N 23° E). Pied flycatchers readily breed in nest boxes enabling efficient manipulation of nest-site availability and breeding monitoring. Experimental design consisted of separate sites (ca. 1 ha in area) each including four nest boxes set-up in a square, at least 60 m apart. Sites were located in pine-dominated forest patches with low availability of natural breeding cavities and did not previously contain nest boxes. Sites were at least a kilometer apart from each other. We set up the box sites in late June 2013 and created four treatments representing different breeding prospects and site quality information for the following breeding season (2014):

1. good breeding quality (real successful great tit nests in all boxes; hereafter ‘Tit’ treatment)
2. suitable quality (nest sites available, but boxes empty; ‘Suitable’ treatment)
3. poor quality, control (unsuitable cavities, boxes upside-down and roofs open; ‘Unsuitable’ treatment)
4. no cavities, control (no boxes at all, empty sites; ‘Empty’ treatment).

Ten replicates of each treatment, i.e., 40 sites, were set up in the Oulu study area and six replicates, i.e., 24 sites, in the Kauhava study area. We randomized each site to one of the four treatments, but so that each subsequent set of four sites included all treatments. The great tit nests were from successful breeding attempts with recently fledged offspring and were collected from nest boxes in nearby study areas maximum 2 days before setting up the treatments. Tit nests are clearly distinguishable from flycatcher nests, because tits use different nest material (moss and hair) than flycatchers (dry hay, grass, and bark). Nest boxes remained in the sites until flycatcher migration, but were removed after that. In both study areas, flycatcher nestlings fledge during the last few weeks of June, the latest ones in early July. The habitat quality information simulated by the treatments was, therefore, available for flycatchers only during the post-breeding season. In the next spring (2014), suitable but empty boxes were put up in all sites regardless of the treatment (also in the ‘Empty’ treatment; in total 160 boxes in Oulu and 96 boxes in Kauhava). Boxes within a site were set up in the same locations as in the previous year. In the ‘Empty’ sites, we used the same box distribution design than in the other treatments.

### Video recording of fall prospecting behavior

We examined whether flycatchers visited the experimental boxes during the post-breeding period in 2013 by using video cameras mounted inside the boxes. Video data consisted of 947 daylight hours recorded between 3rd and 30th of July in 15 nest boxes across the treatments in the Oulu study area. Four different flycatcher individuals, three males (identification based on forehead patch size and shape) and one female, were observed. Total recording time included only 1.3% of total daylight prospecting hours available at nest boxes in July (120 boxes, daylight time in Oulu on average 20 h per day in July). Therefore, we monitored only a fraction of potential prospectors. We could not capture and mark the flycatchers prospecting the experimental nest boxes. Therefore, we did not know which flycatchers settling into the experimental sites in the next spring 2014 had seen the experimental sites in 2013. Most likely some of the settling birds were naive to the information simulated in the sites in the previous year and made their habitat choice randomly in respect of the treatments. This makes our experiment conservative for testing the effects of past information on current breeding site choice. Statistically significant results would thus suggest a strong effect of the simulated past information on flycatcher breeding site choice.

### Breeding season tit surveys and nest monitoring

We controlled for current social information sources (abundance and reproductive investment of conspecifics and heterospecific tits) available during the response year (2014). In May, before setting up the boxes, playback point counts were conducted in the middle of each site to quantify the overall abundance of tits. The playback lasted for 5 min and consisted of great tit singing and warning sounds. Counts were done between 7:00 and 12:00 in fair weather. The number of individuals of all observed tit species (great tit, blue tit *Cyanistes caeruleus*, willow tit *Poecile montanus*, crested tit *Lophophanes cristatus,* and coal tit *Periparus ater*) were recorded, observations were categorized as close or far (less or more than 50 m away) and an index of tit abundance was calculated (see “Data preparation and variable descriptions”). Unavoidably, some of the nest boxes in the response year became occupied by great tits (43 nest boxes; Online Resource 1). To ensure that four boxes were available for flycatchers in each site, a new box was put up nearby for each box occupied by tits.

Flycatcher settlement and breeding were monitored by visiting the boxes and recording the flycatcher nest stage every 2 days until the first egg was laid. To avoid secondary flycatcher females from settling into the sites (a female mated to an already paired male), all empty boxes were removed from a site once the first egg was laid in any of the flycatcher nests in that site. Thus, all pairs that settled, meaning pairs that started building their nest before the first female started egg laying in the site, were allowed to breed. Flycatcher nest building phase takes about 7–9 days (the 25–75% quartile range in the current data set; median 7 days) that is enough time for the great majority of the population to get settled. Removal of nest boxes also was site-specific and egg laying in one site did not affect the availability of nest boxes in other sites. At least one nest box in each treatment was available for settlement in both study areas until the end of the experiment. We restricted the study to primary females, because secondary females receive little assistance from the male, which may affect their breeding decisions and success (Lundberg and Alatalo 1992).

In the late egg laying phase, all (*n* = 82 of 146 clutches) or a sample of flycatcher eggs (at least 4 eggs; usually > 70% of the clutch; *n* = 57) were weighed using digital scale (accuracy 0.01 g). Seven clutches were not weighed due to logistical constraints. Adult females were captured, aged (1 year old, hereafter termed “young” vs. at least 2 years old, hereafter termed “old”; Jenni and Winkler 1994) and measured (tarsus length) during the incubation period. Final clutch size was recorded at the same time. Once nestlings were at least 5 days old, most adult males (116 of 146) were captured, aged, and measured. Brood size was recorded at the time of male catching, and fledgling number was calculated by subtracting from the brood size the number of dead chicks observed in the box after the breeding season. In addition, we followed the settlement and breeding of great tits in the nest boxes in each site to record their abundance, laying date of the first egg, and final clutch size.

### Data preparation and variable descriptions

Response variables to measure flycatcher settlement decisions included nest box occupancy and laying date of the first egg (hereafter laying date). A nest box was defined as occupied when nest material was observed inside the box. In case the nest was already half-built when it was observed for the first time, the box was defined as having been occupied on the previous day. Only nests that proceeded to egg laying were considered. The use of social information may vary between old and young individuals and between females and males (e.g., Doligez et al. 1999; Hahn and Silverman 2006; Parejo et al. 2007b; Forsman et al. 2012; Kivelä et al. 2014; Samplonius and Both 2017). Therefore, in addition to the overall analysis ignoring sex- or age-related differences, nest box occupancy was also examined separately for old and young females and males to see if settlement patterns differ between ages and sexes. Breeding investment and success variables included clutch size, mean egg mass, clutch mass, brood size, and fledgling number. Clutch mass was estimated by multiplying the mean mass of measured eggs (i.e., mean egg mass) with final clutch size.

Present conspecific information sources were estimated as the number of other flycatcher pairs breeding in each site (PFabundance) and their breeding investment (mean egg number; PFeggs). Since we removed all empty boxes from a site once the first flycatcher egg was laid in the site, the PFabundance refers to the number of other flycatcher pairs constructing their nests at the site on the day of nest initiation by the focal pair. For the same reason, no pair was able to settle when other pairs had eggs, and thus, PFeggs was not used as an explanatory variable in nest box occupancy analyses. Heterospecific information sources were estimated as the presence or absence of breeding great tits in each site (GTpresence) and mean egg number of all great tits in the site (GTeggs). To control temporal variability in these information sources (number of pairs and eggs naturally increase along the time), values for these variables were derived for each day within the flycatcher settlement period. In addition to these measures of con- and heterospecific social information sources, we used the survey data collected at the beginning of the breeding season to calculate an abundance index of all tit species. The index was calculated as a weighed sum of all observed individuals, the weights being 1 for close and 0.5 for far observations (hereafter TitIndex). TitIndex and GTpresence both describe the abundance of heterospecific tits, but their impact on flycatcher breeding may be different. TitIndex measures total tit abundance (i.e., five tit species combined) and may represent general information about site quality or the amount of interspecific competition in a site. GTpresence instead may represent a more specific source of information for flycatchers, for example on nest-site availability and quality, and may be more easily perceived by flycatchers, since both species breed in the same kind of nest boxes.

### Statistical analyses

Nest box occupancy was analyzed using mixed Cox proportional-hazard regression models (further details in Online Resource 1). The rest of the response variables were analyzed using generalized linear mixed models (GLMM) with Gaussian (laying date, mean egg mass, and clutch mass) or Poisson (clutch and brood size, fledgling number) error distribution. Nest box (Cox models) or nest (GLMMs) was used as the observational unit and site was included as a random effect. In GLMMs, the values of time-dependent variables, i.e., variables related to great tits and other flycatchers, were set to represent the situation on the day when the first egg was laid. Cox models readily incorporate time-dependent variables, and thus, great tit and flycatcher presence/abundance and investment variables had day-specific values in the nest box occupancy analysis.

Full models varied between different response variables and data sets due to varying availability of explanatory variables and data limitations, but in general all available, relevant variables were included (see Table A1 in Online Resource 1 for the full models). The maximum set of variables included Treatment (Tit, Suitable, Unsuitable, Empty), Area (Kauhava, Oulu), TitIndex, great tit, and conspecific abundance (GTpresence, PFabundance) and investment (GTeggs, PFeggs), and phenotypes of female and male flycatcher adults (age, tarsus length). Also two-way interactions between treatment and other variables were included if there were at least 15 observations per treatment for continuous variables or 20 observations for a categorical variable (ten for each category). The interactions were included to test for varying treatment effects between the study areas and to control for the effects of availability of other social information sources (see, e.g., Jaakkonen et al. 2015; Firth et al. 2016) and of individual’s phenotype (age and body size; see, e.g., Forsman et al. 2012; Loukola et al. 2013; Kivelä et al. 2014; Tolvanen et al. 2018) on the use of past information sources, the treatments.

Cox proportional-hazard regression models assume proportional hazards; that is, that the ratio of hazards (probability of occupancy) between observational units (nest boxes) is constant over time. We tested this assumption for all full models using the function *cox.zph* in R package *Survival* and excluded the explanatory variables or interactions that did not pass the test (*p* < 0.05). The resulting full models fulfilled the assumption (global tests, *p* > 0.22). Also all the final models (see below) fulfilled the proportionality assumption (global tests, *p* > 0.32).

After defining the full model, all models under the full model, but retaining the treatment and random effect, were fitted. Akaike’s information criterion (Akaike 1974) corrected for small-sample size, AICc, was used to rank the models. To take model selection uncertainty into account, we derived final model sets that included all models with ΔAICc < 6, but with the constraint that models that were more complex versions of a model with lower AICc were omitted (Richards et al. 2011). If more than one model was included in the final model set, we evaluated the relative support between the models using evidence ratios (ratios of model Akaike weights; Burnham and Anderson 2002). Evidence ratio indicates how likely the better model in the current analysis would remain as the better model if the experiment was repeated (Burnham and Anderson 2002). Inferences were based on the evidence ratios and parameter effect sizes and their 95% confidence intervals (95% CI) in the best supported models. Our principal interest was in the treatment effects; therefore, we report all pair-wise comparisons between the treatments (six comparisons). If there were no clear differences between the two control treatments (‘Unsuitable’ and ‘Empty’), that is, if the 95% CI of the difference included zero, we combined the treatments to a single ‘Controls’ treatment. In case the final model used for inferences included an interaction between the treatment effect and a continuous variable, we derived pair-wise treatment comparisons for mean, minimum, and maximum (with observations in all treatments) values of the continuous variable. Analyses were done using program R (version 3.3.1; R Core Team 2016).

Full data sets for each response variable consisted of nests for which female information was available. Nine nests (four in Oulu; five in Kauhava) were abandoned before we could capture the females and were thus omitted from analyses, except for the overall nest box occupancy analyses where adult information was not needed (all nests progressing to egg laying included). We also split data according to the presence of breeding great tits in the site (GT data) or not (NoGT data). First, we wanted to control for the impact of heterospecific (great tit) breeding investment (egg number) during the current breeding season on flycatcher breeding decisions. Second, we wanted to test how flycatcher response to treatments was affected by the availability of social information from currently breeding great tits (see Jaakkonen et al. 2015; Firth et al. 2016 for how information availability may affect conspecific vs. heterospecific information use). Furthermore, the effect of conspecific reproductive investment (egg number) was examined using a subsample of sites where at least one flycatcher pair, in addition to the focal pair, was breeding (sites with and without breeding great tits combined to reach adequate sample sizes). Adult male phenotype (age and tarsus length) effects were investigated with a subsample of nests according to trapping success.

The statistical procedure regarding the different subsetted data was started by testing the effects of male phenotype, but it did not have a considerable impact on any response variable (results not shown). We then proceeded to test the effect of conspecific reproductive investment, but again, the effect was not important regarding any response variable (results not shown). Therefore, the inferences were based on the analyses of GT and NoGT data sets or, if the results did not differ between them, on the full data set. For the brood size and fledgling number analyses, we used only the data including also male information, i.e., nests where both parents were surely brooding. Summaries of the analyzed response variables, used subset data sets, full models, and sample sizes are presented in Table A1 (Online Resource 1).

## Results

### Nest box occupancy

**Fig. 1:**
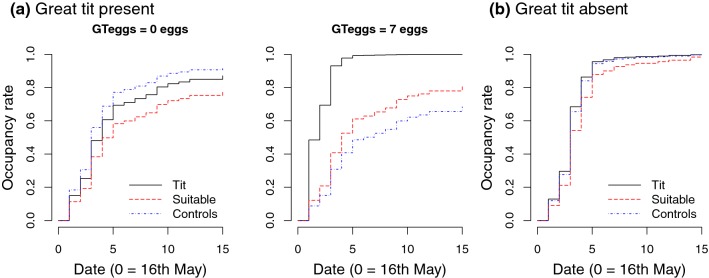
Pied flycatcher occupancy patterns **a** in sites where great tits (GT) were breeding (model predictions with GT egg number fixed to 0 vs. 7 eggs) and **b** in sites where great tits were absent relative to different treatments. Different types of lines depict the predicted cumulative occupancy rates in different treatments across the settlement period, based on the best supported model in each data set. Sample sizes in **a**: Tit, *n* = 34; Suitable, *n* = 38; Controls, *n* = 60 nest boxes; and in **b**: Tit, *n* = 40; Suitable, *n* = 29; Controls, *n* = 100 nest boxes. The color version of the figure is available online

Overall, 155 of 250 nest boxes (62.0%) were occupied by pied flycatchers (Online Resource 1). Half of captured females (83 of 146 females; 56.8%) and males (63 of 116 males; 54.3%) were old birds (at least 2 years old). Settlement patterns differed depending on whether breeding great tits were present or not at the sites. For sites including breeding great tits, the final model set included four models with the first two models clearly better supported than the third and fourth models [evidence ratio (ER) between the first model and the third model, ER = 16.0; Table 1]. The first model and the second model were equally supported (ER = 1.0, Table 1). Both models indicated higher occupancy rate with increasing conspecific abundance in the site (for the first model: *β* = 0.38, 95% CI 0.06–0.70; Table A2 in Online Resource 1) and lower occupancy rate in Oulu than in Kauhava (for the first model: *β* = − 0.91, 95% CI − 1.52 to − 0.30; Table A2 in Online Resource 1). The models differed in that the first model included the effect of the number of great tit eggs (GTeggs) at the time of flycatcher settlement and an interaction between treatment and GTeggs, but the second model excluded both (Table 1). The first model indicated no occupancy differences between the treatments when great tit egg number was zero, but increasing preference of the Tit treatment compared to the other treatments with increasing number of tit eggs (GTeggs range 0–10 eggs; Fig. 1a; Table A2 in Online Resource 1). At the average tit egg number (2.6 eggs), occupancy rate was clearly higher in the Tit treatment than in the Suitable treatment (*β* = 0.79, 95% CI 0.07–1.51). When the tit egg number was seven eggs (maximum egg number in the data with observations in all treatments), occupancy rate was clearly higher in the Tit treatment than in the Suitable (*β* = 1.63, 95% CI 0.03–3.22) and in the Controls (*β* = 1.98, 95% CI 0.56–3.39; Fig. 1a.). Based on the second model, occupancy rate was higher in the Tit treatment than in the Suitable treatment (*β* = 0.67, 95% CI 0.01–1.32).

**Table 1:** Statistics of the final model sets

Response variable	Data set	Model	*df*	AICc	ΔAICc	Akaike weight
Occupancy	GT sites	Treatment + Area + GTeggs + PFabundance + Treatment × GTeggs	7.02	577.62	0.00	0.48
		Treatment + Area + PFabundance	4.02	577.72	0.10	0.46
		Treatment + TitIndex + PFabundance	4.02	582.89	5.27	0.03
		Treatment + PFabundance	3.03	583.41	5.78	0.03
	No GT sites	Treatment + Area + PFabundance	4.01	688.10	0.00	0.58
		Treatment + Area	3.01	688.76	0.66	0.42
	Old females	Treatment + Area	3.03	789.38	0.00	1.00
	Young females	Treatment + Area + Treatment × Area	10.52	605.86	0.00	1.00
	Old males	Treatment + Area + PFabundance	4.02	572.80	0.00	0.94
		Treatment + Area	5.49	578.35	5.54	0.06
	Young males	Treatment	2.02	519.17	0.00	1.00
Laying date	Female	Treatment + Area	6	746.44	0.00	1.00
Clutch size	Female	Treatment + LayingDate	5	567.18	0.00	0.56
		Treatment	4	567.67	0.49	0.44
Egg mass	Female GT	Treatment + TitIndex + ClutchSize + FemaleAge + Treatment × TitIndex	10	− 95.23	0.00	0.47
		Treatment + TitIndex + FemaleAge + Treatment × TitIndex	9	− 94.17	1.05	0.28
		Treatment + ClutchSize + FemaleAge	7	− 92.55	2.68	0.12
		Treatment + FemaleAge	6	− 90.77	4.46	0.05
		Treatment + TitIndex + Treatment × TitIndex	8	− 90.52	4.71	0.04
		Treatment	5	− 90.19	5.04	0.04
	Female no GT	Treatment + LayingDate	6	− 105.58	0.00	0.56
		Treatment	5	− 105.06	0.52	0.44
Clutch mass	Female	Treatment + FemaleAge + LayingDate	7	493.27	0.00	1.00
Brood size	Adult	Treatment + ClutchSize	5	424.61	0.00	1.00
Fledgling number	Adult	Treatment + ClutchSize	5	437.58	0.00	1.00

Only the data sets on which the final inferences were based are included

In sites without breeding great tits, the final model set included two models (Table 1) consistently indicating no differences between the treatments (Fig. 1b; Table A2 in Online Resource 1). In contrast to the sites where great tits were breeding, occupancy rate was not positively associated with conspecific abundance, and even appeared to decrease with increasing conspecific abundance in the site, but the 95% CI of the mean estimate included zero (*β* = − 0.27, 95% CI − 0.60 to 0.06; ER = 1.4 between the first model and the second model excluding the PFabundance effect). The occupancy rate difference between the study areas was even stronger than in the sites where great tits were breeding, with lower occupancy in Oulu (*β* = − 1.95, 95% CI − 2.58 to − 1.32). For the mean estimates and their 95% CIs of the other parameters not mentioned here, see Table A2 in Online Resource 1.

**Fig. 2:**
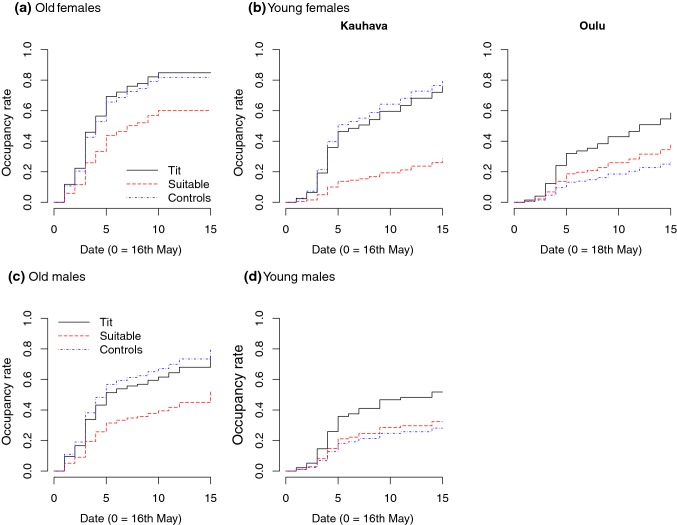
Occupancy patterns of **a** old female, **b** young female, **c** old male, and **d** young male pied flycatchers in different treatments. For young females, the patterns differed between the study areas, Kauhava and Oulu. Different types of lines depict the predicted cumulative occupancy rates in different treatments across the settlement period, based on the best supported model in each data set. Sample sizes in **a**: Tit, *n* = 67; Suitable, *n* = 67; Controls, *n* = 139 nest boxes; in **b**: Kauhava: Tit, *n* = 24; Suitable, *n* = 26; Controls, *n* = 52 nest boxes; Oulu: Tit, *n* = 43; Suitable, *n* = 41; Controls, *n* = 87 nest boxes; and in **c** and **d**: Tit, *n* = 65; Suitable, *n* = 67; Controls, *n* = 135 nest boxes. The color version of the figure is available online

For the occupancy rate of old females (sites with and without breeding great tits combined), the final model (Table 1) indicated higher occupancy rate in the Tit treatment than in the Suitable treatment (*β* = 0.72, 95% CI 0.07–1.37; Table A3 in Online Resource 1) and lower occupancy rate in the Suitable treatment than in the Controls (*β* = − 0.62, 95% CI − 1.20 to − 0.04; Fig. 2a). Occupancy rate of old females was lower in Oulu than in Kauhava (*β* = − 1.68, 95% CI − 2.16 to − 1.20). For the occupancy rate of young females, the final model (Table 1) indicated that the treatment effects differed between the study areas (Table A3 in Online Resource 1). In Kauhava, occupancy rate was higher in the Tit treatment than in the Suitable treatment (*β* = 1.45, 95% CI 0.01–2.90) and lower in the Suitable treatment than in the Controls (*β* = − 1.60, 95% CI − 2.91 to − 0.29; Fig. 2b). In Oulu, occupancy rate was higher in the Tit treatment than in the Controls (*β* = 1.02, 95% CI 0.18–1.86; Fig. 2b; Table A3 in Online Resource 1).

Male age was not clearly associated with the age of the female within a pair (GLM, female age as the binary response variable (old = 1) and male age as the predictor: male age, young *β* = − 0.36, 95% CI − 1.12 to 0.38). Therefore, the settlement patterns of old and young females and males seem independent of each other. For occupancy rate of old males, the final model set consisted of two models with the first model clearly better supported than the second one (ER = 15.7; Table 1). The first model indicated lower occupancy rate in the Suitable treatment than in the Controls (*β* = − 0.80, 95% CI − 1.51 to − 0.09; Fig. 2c). Occupancy rate of old males also increased with increasing conspecific abundance (*β* = 0.41, 95% CI 0.06–0.76) and was lower in Oulu than in Kauhava (*β* = − 1.65, 95% CI − 2.29 to − 1.00; Table A3 in Online Resource 1). The final model for the occupancy rate of young males (Table 1) indicated higher occupancy rate in the Tit treatment than in the Controls (*β* = 0.79, 95% CI 0.14–1.44; Fig. 2d). For the mean estimates and their 95% CIs of the other parameters not mentioned here, see Table A3 in Online Resource 1.

### Timing of breeding, reproductive investment, and success

For timing of breeding (laying date of the first egg), the final model (Table 1) indicated clearly later laying date in the Suitable treatment than in the Controls (*β* = 2.01, 95% CI 0.64–3.37) and in Oulu than in Kauhava (*β* = 3.47, 95% CI 2.40–4.53; Tables A4 and A5 in Online Resource 1). For clutch size, the final model (Table 1) suggested no differences between treatments (Table A5 in Online Resource 1).

The treatment effects on mean egg mass differed between sites where great tits were currently breeding and sites where they were absent. For sites where great tits were currently breeding, the final model set included six models of which the first two were better supported than the rest of the models (ER = 3.9 between the first model and the third model; Table 1). Both the first model and the second model included an interaction between treatment and overall tit abundance (TitIndex) and differed only by the exclusion of clutch size effect in the second model (Table 1). Mean egg mass decreased with higher overall tit abundance in the Suitable treatment (*β* = − 0.05, 95% CI − 0.10 to − 0.01), but not so in Tit (*β* = 0.05, 95% CI − 0.01 to 0.11) and Controls treatments (*β* = 0.02, 95% CI − 0.003 to 0.04; Table A5 in Online Resource 1). When tit abundance was zero, mean egg mass was lower in the Tit treatment than in the Suitable treatment (*β* = − 0.11, 95% CI − 0.21 to − 0.01) and higher in the Suitable treatment than in the Controls (*β* = 0.13, 95% CI 0.03–0.22). With increasing tit abundance, mean egg mass estimates were highest for the Tit treatment, but the differences were not clear (TitIndex = 2: Tit vs. Suitable, *β* = 0.10, 95% CI − 0.01 to 0.22; Tit vs. Controls, *β* = 0.09, 95% CI − 0.01 to 0.19; Table A5 in Online Resource 1). Mean egg mass also was lower in young females than in old females (*β* = − 0.09, 95% CI − 0.15 to − 0.03). In sites where great tits were not currently breeding, there were no differences in mean egg mass between the treatments (Table A5 in Online Resource 1).

The results for clutch mass, representing total breeding investment (mean egg mass multiplied by clutch size), did not differ between sites where great tits were currently breeding and sites where they were absent. The final model (Table 1) suggested no differences between treatments (Tables A4 and A5 in Online Resource 1), but lower clutch mass in young than in old females (*β* = − 0.99, 95% CI − 1.48 to − 0.50) and the later the egg laying was started (*β* = − 0.11, 95% CI − 0.18 to − 0.03).

Brood size and fledgling number final models (Table 1) indicated no differences between treatments (Tables A4 and A5 in Online Resource 1), but a positive clutch size effect in both (*β* = 0.15, 95% CI 0.07–0.23 for brood size and *β* = 0.12, 95% CI 0.04–0.20 for fledgling number; Table A5 in Online Resource 1). For the mean estimates and their 95% CIs of the other parameters not mentioned here, see Table A5 in Online Resource 1.

## Discussion

Pied flycatcher settlement behavior was affected by the experimentally simulated habitat quality and nest-site availability information collected during the post-breeding period of the previous year. Our results show that flycatchers prospect nesting areas during the post-breeding period and extract information based on potential nest sites and nest remains. Also, timing of breeding and reproductive investment measured as mean egg mass were influenced by the information cues available in the previous post-breeding season. Flycatchers also relied on social information sources present during the settlement and, in some cases, appeared to integrate both past and present information sources suggesting sequential updating of social information in breeding decisions. Differences between the study areas were limited to the occupancy patterns of young females, suggesting that the information use strategies are relatively similar across the geographical areas.

Flycatcher settlement patterns differed depending on whether great tits were currently breeding in the site or not. In sites where great tits were simultaneously breeding in nest boxes, flycatchers preferred the sites where tits had apparently been breeding in the previous year (Tit treatment) compared to other sites, but only when great tit reproductive investment (number of eggs) was relatively high. This suggests that birds can combine past and current information sources in their settlement decisions and copy previous habitat choices of heterospecifics if heterospecifics also show high performance during the settlement year. However, the best model was not clearly better than the second-best model that excluded the effect of great tit reproductive investment. The second-best model suggested that flycatchers preferred the Tit treatment sites to those where nest sites were available but empty in the previous year (Suitable treatment), but the difference to control sites was not clear. In sites without currently breeding great tits, flycatcher settlement did not differ between the treatments. In sites with breeding great tits, flycatchers also preferred sites with higher conspecific abundance, whereas the pattern appeared the opposite albeit not clearly so in sites without great tits. Birds thus seem to combine heterospecific and conspecific information sources, so that individuals settling in sites including heterospecifics (potential heterospecific attraction) also show conspecific attraction, whereas those that avoid heterospecifics may also avoid conspecifics.

Our results showed that all sex and age groups used the simulated information cues present in the previous post-breeding season. However, we did not observe a consistent preference for any one treatment. For young females, the occupancy patterns differed between the study areas; in Kauhava, they avoided the Suitable treatment, whereas in Oulu, they preferred the Tit treatment over the Control sites. Young males tended to especially prefer the Tit treatment, with comparable differences to the Controls (mean = 0.79, 95% CI [0.14–1.44]) and the Suitable treatment (0.63, [− 0.08 to 1.33]), although the latter estimate was less precise and not clearly different from zero. By cueing on nest cavity contents in the post-breeding season and then copying the site choice of heterospecifics in the next spring, young flycatchers may enhance their chances of breeding in good-quality environment. This may be particularly useful for young birds which do not have previous breeding experience and thus lack own knowledge about breeding site choice and its consequences. Young birds also tend to arrive in breeding grounds later than older individuals (Potti 1998; Both et al. 2016) making them more time constrained in breeding site choice. Making the site choice already in the previous year should make the settlement faster in their first spring and thus ameliorate the time constraint and facilitate successful breeding.

An alternative explanation for preferring the sites used by other birds in the previous year could be to get nest construction benefits by building the nest on top of the old nest remains (Loukola et al. 2014). This is, however, an unlikely explanation, because it would not explain why preference for the Tit treatment was apparent only if great tits were also currently breeding in the site and had a high number of eggs, or that such preference was mainly observed in young (except for young females in Kauhava) but not in old flycatchers (see discussion below). Nest construction benefits should be equal for all flycatchers irrespective of current abundance or breeding investment of great tits or individual age. Indeed, all flycatchers, regardless of their age, strongly prefer nest boxes containing some material to empty nest boxes (Loukola et al. 2014). Also, all the nest boxes provided during the flycatcher settlement were empty, and thus, no nest construction benefits were available anymore. Flycatchers use various information sources during settlement to make their breeding site choices (see results here and discussion and references in the “Introduction”); therefore, it seems unlikely that past information about potential nest construction benefits that is no longer available during the settlement would have a considerable effect in breeding site choice.

In contrast to the general pattern in young flycatchers, old flycatchers did not prefer sites where nest boxes contained tit nests in the previous year but avoided sites where nest boxes were available but empty (the Suitable treatment). The avoidance of the Suitable treatment is somewhat surprising, since birds could be expected to also prefer sites where there were suitable breeding cavities available, especially since tree cavities are generally in short supply in managed forests. Avoidance of nest sites not chosen by any other bird may, nevertheless, be adaptive, because empty nest sites indicate that the site is otherwise of very low quality. The later timing of breeding in the Suitable treatment may also be a sign of avoiding sites not chosen by other birds in the previous year until late in the season when other, better sites have perhaps already been occupied. These results are consistent with earlier studies reporting lower occupancy rate or later timing of breeding in nest boxes lacking old nest remains (Davis et al. 1994; Loukola et al. 2014; Sumasgutner et al. 2014), although these studies did not differentiate between avoidance of empty nest boxes and attraction to nest boxes including nest remains.

From measures of reproductive investment, clutch size and clutch mass (total reproductive investment) did not differ between the treatments. Mean egg mass, however, was influenced by the combined effect of the treatments and current overall tit abundance in the site. When tit abundance was low, mean egg mass was higher in the Suitable treatment than in the other treatments. With increasing tit abundance, the pattern changed to indicate higher egg mass in the Tit treatment compared to the other treatments, although the differences were not clear. Moreover, these patterns in egg mass were only present for flycatchers breeding in sites, where also great tits were simultaneously breeding. In sites where great tits were absent, there were no differences in mean egg mass between treatments. This is the second indication in our study that suggests sequential information use in flycatcher breeding decisions. Here, past social cues (nest remains of breeding heterospecifics) seem to lead to an increase in mean egg mass but only if great tits also currently breed in the site and if the tit abundance in overall (all tit species combined) is relatively high. Such information use pattern parallels that observed in the nest box occupancy analysis where the Tit treatment was preferred if great tits were also currently breeding and had relatively high number of eggs. The higher egg mass in the Suitable treatment when tit abundance was low is a bit surprising. If the birds perceived the Suitable treatment sites as particularly bad-quality sites (see discussion above), then those birds still settling in these sites may have tried to compensate for the low apparent quality of the environment by laying larger eggs. Larger eggs usually result in better quality offspring (Krist 2011) that may survive despite the rearing environment is more challenging.

Updating information based on past cues with information based on more recently available cues makes sense, because the value of information decreases as the temporal distance between collecting and implementing it in decision-making increases (Seppänen et al. 2007; Thomson et al. 2013; Tolvanen et al. 2018). Such sequential updating of information, often termed as Bayesian information updating (Valone 1989, 2006), has been suggested to be adaptive by theoretical studies (Green 1987; Olsson and Brown 2010; Szymkowiak and Kuczynski 2015), but empirical evidence is scarce (Valone 2006; Schmidt et al. 2010). Foraging animals have been observed to update personal information across time (Valone and Brown 1989; Vásquez et al. 2006; Marshall et al. 2013) and to combine personal with social information (Kendal et al. 2004; Dawson and Chittka 2014; Heinen and Stephens 2016). In breeding habitat choice, multiple types of personal and/or social information sources have been observed to be concurrently used at the population level (e.g., Sergio and Penteriani 2005; Redmond et al. 2009; Kivelä et al. 2014), but whether it is the same or different individuals using the multiple information sources remained unknown (but see Boulinier et al. 2008 for an example of combining personal and social information). Our results suggest that animals may sequentially update one or more types of social information.

Brood size and number of fledglings did not differ between the treatments or in relation to any other social cue considered. This suggests that habitat quality did not notably differ between the treatments and did not correlate, for example, with the number of great tit eggs, conspecific abundance, or the overall tit abundance in the site. Thus, the observed occupancy and reproductive investment patterns most likely demonstrate the use of social information in breeding decisions.

In conclusion, our experiment provides convincing evidence that nest remains of heterospecifics serve as an additional social information source for animals, but that the use of such information may vary between individuals. The ability to perceive nest remains as information considerably increases available social information. Such information is available as long as the old nests remain observable (potentially throughout the post-breeding period, even until the next spring), and not only for the adults and fledglings from the surrounding areas, but also for dispersing or migrating individuals passing through the area. Moreover, past social information cues present in the previous post-breeding season appeared to be updated by more recent cues during the settlement period, in a way that suggested sequential social information use. Our results highlight the diverse nature of information use in animal decision-making, and hopefully serve as a starting point for further investigations of sequential information use strategies, both in general and in relation to individual-level variation within them.

## Electronic supplementary material

Below is the link to the electronic supplementary material.

Supplementary material 1 (PDF 330 kb)

## Data Availability

The data generated during the current study are available in the Dryad repository: 10.5061/dryad.5tb2rbp1m.
