# Supplementary material for: Information collected during the post-breeding season guides future breeding decisions in a migratory bird
Source: Oecologia. 2020 Mar 12;192(4):965–77. doi: 10.1007/s00442-020-04629-5 (PMC7165145; doi:10.1007/s00442-020-04629-5)
Supplement: Supplementary file 1 — Supplementary material 1 (PDF 330 kb) [file 442_2020_4629_MOESM1_ESM.pdf]

Online Resource 1; Supplementary material

Information collected during the post-breeding season guides future breeding decisions in a migratory bird

Jere Tolvanen, Chiara Morosinotto, Jukka T. Forsman & Robert L. Thomson

Corresponding author:
Jere Tolvanen
Email: jeretolvanen87@gmail.com

Supplementary details of the methods

Flycatchers compete with tits over nest sites and occasionally take-over tit nest sites (pers. obs.). We omitted such occasions from the analyses (three nests in Oulu data, one nest in Kauhava data), since they may not be comparable to the usual behavior of settling in an unoccupied nest site. One nest with unknown nest initiation date was also omitted from the Oulu data. However, all nests progressing to egg laying (including the takeovers and the nest with unknown nest initiation date) were taken into account in defining conspecific and great tit abundance and investment variables (PFabundance, PFeggs, GTpresence and GTeggs).

Cox regression models have been traditionally used in medical disciplines and survival analyses, but are readily extended to any time-to-event data (Cox and Oakes 1984; Therneau 2012). In this study the event was the occupancy of a nest box. Cox models enable efficient use of the data, when the timing of entering and/or leaving the study differs between observational units and when some variables of interest are time-dependent, as is the case in this study. For example, new nest boxes were put up for each box occupied by great tits and non-occupied nest boxes were removed once the first flycatcher egg was laid in a site resulting in both left- and right-censored data (i.e. the timing of both entering and leaving the study varies between nest boxes). In addition, the variables describing current conspecific and heterospecific information sources varied temporally due to increasing number of birds settling into the sites and due to progress in egg laying as the breeding season continued. Cox models make it possible to use temporally variable covariates, because a unique variable value can be assigned to each day during the settlement period (the number of rows for each nest box in the data set equals the number of days the box is available for settlement).

In some analyses additional variables potentially explaining the variation in response variables were included to enable more precise analyses. Timing of breeding was controlled for by including the laying date as a covariate in the breeding investment and success analyses. Clutch size was included as a covariate in the analyses of mean egg mass, and both clutch size and mean egg mass in the analyses of brood size and fledgling number. Preliminary data exploration was done in order to identify potential non-linear effects of continuous variables, but no non-linear trends were observed. Continuous explanatory variables were mean-centered (to overall data set-specific means) prior to analyses. Collinearities of continuous explanatory variables were estimated using Pearson correlation coefficients. Highly collinear continuous explanatory variables ($r > 0.6$) were not allowed in the same model.

Because, to our knowledge, there is no simple way to make figures based on mixed Cox regression models, we illustrate the flycatcher settlement patterns using figures based on the models without the random effect (i.e. ordinary Cox regression). This approach nevertheless provides an accurate illustration of the results of the mixed models, since the coefficient estimates of the fixed effects in the models fitted without the random effect were qualitatively identical and also quantitatively very close to the mixed model coefficient estimates.

The specific R packages used in the analyses included 'coxme' (Therneau 2014), 'nlme' (Pinheiro et al. 2015), 'lme4' (Bates et al. 2015) and 'MuMIn' (Bartoń 2014).

Supplementary details of the results

In Oulu the flycatcher settlement period lasted 15 days (19th May-2nd June). Seventy of 155 nest boxes (45.2%) were occupied by pied flycatchers. Less than half of captured females (29 of 66 females; 43.9%) and a third of males (21 of 58 males; 36.2%) were old birds (at least 2-years-old). Great tits occupied at least one nest box in half of the study sites (19 of 40 sites) across all four treatments (six Tit treatment sites, three Suitable sites, five Unsuitable sites and five Empty sites). The overall abundance index of tits (TitIndex) was on average 1.2 (range 0–5); in 20 sites the TitIndex was 0–0.5 (practically no tits present) and in the other 20 sites 1–5. Treatment-specific mean TitIndexes were 0.8 in Tit, 0.9 in Suitable, 2.1 in Unsuitable and 1.2 in Empty.

In Kauhava the settlement period lasted 10 days (17th-26th May). Eighty-five of 95 nest boxes (89.5%) were occupied by flycatchers. Two thirds of captured females (54 of 80 females, 67.5%) and males (42 of 58 males, 72.4%) were old birds. Great tits occupied at least one nest box in 14 of

24 study sites (three Tit sites, six (all) Suitable sites, three Unsuitable sites and two Empty sites). Mean TitIndex was in overall 2.1 (range 0–7) and treatment-specifically 1.2 in Tit, 1.2 in Suitable, 4.3 in Unsuitable and 2.0 in Empty.

References

Bartoń K (2014) Package ‘MuMIn’: Multi-model inference. Version 1.10.5 (CRAN)

Bates D, Maechler M, Bolker B, Walker S, Christensen RHB, Singmann H, Dai B (2015) Package ‘lme4’: Linear mixed-effects models using Eigen and S4. Version 1.1-7 (CRAN)

Cox DR, Oakes D (1984) *Analysis of survival data*. Chapman and Hall, New York

Pinheiro J, Bates D, DebRoy S, Sarkar D, EISPACK authors, R-core (2015) Package 'nlme': Linear and nonlinear mixed effects models. Version 3.1-119 (CRAN)

Therneau T (2012) Mixed effects Cox models. Available: https://cran.r-project.org/web/packages/coxme/vignettes/coxme.pdf. Accessed 7th November 2014

Therneau T (2014) Package 'coxme': mixed effects Cox models. Version 2.2-3 (CRAN)

Tables including the response variables, data sets, full models and sample sizes

Table A1. Response variables, data sets, full models (fixed effects structure) and sample sizes for the pied flycatcher data.

**Response variable**	**Data set**	**Full model (Fixed effects)**	**n**
Nest box occupancy	GT, all birds	Treatment + Area + TitIndex + GTeggs + PFabundance + Treatment*Area + Treatment*TitIndex + Treatment*GTeggs + Treatment*PFabundance	132 boxes
	NoGT, all birds	Treatment + Area + TitIndex + PFabundance + Treatment*TitIndex + Treatment*PFabundance	169 boxes
	Old females	Treatment + Area + TitIndex + GTpresence + PFabundance + Treatment*Area + Treatment*TitIndex + Treatment*GTpresence + Treatment*PFabundance	273 boxes
	Young females	Treatment + Area + TitIndex + GTpresence + PFabundance + Treatment*Area + Treatment*TitIndex + Treatment*PFabundance	273 boxes
	Old males	Treatment + Area + TitIndex + GTpresence + PFabundance + Treatment*Area + Treatment*TitIndex + Treatment*GTpresence + Treatment*PFabundance	267 boxes
	Young males	Treatment + Area + TitIndex + GTpresence + PFabundance + Treatment*Area + Treatment*TitIndex + Treatment*PFabundance	267 boxes
Laying date	Female	Treatment + Area + TitIndex + GTpresence + FemaleAge + FemaleTarsus + Treatment*Area + Treatment*TitIndex + Treatment*FemaleAge + Treatment*FemaleTarsus	146 nests
	FemaleGT	Treatment + Area + TitIndex+ FemaleAge + FemaleTarsus + Treatment*TitIndex + Treatment*FemaleTarsus	77 nests
	FemaleNoGT	Treatment + Area + TitIndex + FemaleAge + FemaleTarsus	69 nests
	Adult	Treatment + Area + TitIndex + GTpresence + FemaleAge + FemaleTarsus + MaleAge + MaleTarsus + Treatment*Area + Treatment*TitIndex + Treatment*FemaleAge + Treatment*FemaleTarsus + Treatment*MaleAge + Treatment*MaleTarsus	116 nests
Clutch size	Female	Treatment + Area + TitIndex + GTpresence + PFabundance + LayingDate + FemaleAge + FemaleTarsus + Treatment*Area + Treatment*TitIndex + Treatment*PFabundance + Treatment*FemaleAge + Treatment*FemaleTarsus	145 nests
	FemaleGT	Treatment + Area + TitIndex + GTeggs + PFabundance + LayingDate + FemaleAge + FemaleTarsus + Treatment*TitIndex + Treatment*GTeggs +Treatment*PFabundance + Treatment*FemaleTarsus	73 nests
	FemaleNoGT	Treatment + Area + TitIndex + PFabundance + LayingDate + FemaleAge + FemaleTarsus	69 nests
	FemalePF	Treatment + Area + TitIndex + GTpresence + PFabundance + PFeggs + LayingDate + FemaleAge + FemaleTarsus + Treatment*Area + Treatment*TitIndex + Treatment*PFabundance + Treatment*PFeggs + Treatment*FemaleAge + Treatment*FemaleTarsus	132 nests
	Adult	Treatment + Area + TitIndex + GTpresence + PFabundance + LayingDate + FemaleAge + FemaleTarsus + MaleAge + MaleTarsus + Treatment*Area + Treatment*TitIndex + Treatment*PFabundance + Treatment*FemaleAge + Treatment*FemaleTarsus + Treatment*MaleAge + Treatment*MaleTarsus	116 nests

Table A1 (*continued*). Response variables, data sets, full models (fixed effects structure) and sample sizes for the pied flycatcher data.

Response variable	Data set	Full model (fixed effects)	n
Egg mass	Female	Treatment + Area + TitIndex + GTpresence + PFabundance + LayingDate + ClutchSize + FemaleAge + FemaleTarsus + Treatment*Area + Treatment*TitIndex + Treatment*PFabundance + Treatment*FemaleAge + Treatment*FemaleTarsus	138 nests
	FemaleGT	Treatment + Area + TitIndex + GTeggs + PFabundance + LayingDate + ClutchSize + FemaleAge + FemaleTarsus + Treatment*TitIndex + Treatment*GTeggs + Treatment*PFabundance + Treatment*FemaleTarsus	68 nests
	FemaleNoGT	Treatment + Area + TitIndex + PFabundance + LayingDate + ClutchSize + FemaleAge+FemaleTarsus	67 nests
	FemalePF	Treatment+Area+TitIndex+ GTpresence + PFabundance + PFeggs + LayingDate + ClutchSize + FemaleAge + FemaleTarsus + Treatment*Area + Treatment*TitIndex + Treatment*PFabundance + Treatment*PFeggs + Treatment*FemaleAge + Treatment*FemaleTarsus	125 nests
	Adult	Treatment + Area + TitIndex + GTpresence + PFabundance + LayingDate + ClutchSize + FemaleAge + FemaleTarsus + MaleAge + MaleTarsus + Treatment*Area + Treatment*TitIndex + Treatment*PFabundance + Treatment*FemaleAge +Treatment*FemaleTarsus + Treatment*MaleTarsus	110 nests
Clutch mass	Female	Treatment + Area + TitIndex + GTpresence + PFabundance + LayingDate + FemaleAge + FemaleTarsus + Treatment*Area + Treatment*TitIndex + Treatment*PFabundance + Treatment*FemaleAge + Treatment*FemaleTarsus	138 nests
	FemaleGT	Treatment + Area + TitIndex + GTeggs + PFabundance + LayingDate + FemaleAge + FemaleTarsus + Treatment*TitIndex + Treatment*GTeggs + Treatment*PFabundance + Treatment*FemaleTarsus	68 nests
	FemaleNoGT	Treatment + Area + TitIndex + PFabundance + LayingDate + FemaleAge + FemaleTarsus	67 nests
	FemalePF	Treatment + Area + TitIndex + GTpresence + PFabundance + PFeggs + LayingDate + FemaleAge + FemaleTarsus + Treatment*Area + Treatment*TitIndex + Treatment*PFabundance + Treatment*PFeggs + Treatment*FemaleAge + Treatment*FemaleTarsus	125 nests
	Adult	Treatment + Area + TitIndex + GTpresence + PFabundance + LayingDate + FemaleAge + FemaleTarsus + MaleAge + MaleTarsus + Treatment*Area + Treatment*TitIndex + Treatment*PFabundance + Treatment*FemaleAge + Treatment*FemaleTarsus + Treatment*MaleTarsus	110 nests
Brood size and fledgling number	Adult	Treatment + Area + TitIndex + GTpresence + PFabundance + LayingDate + ClutchSize + MeanEggMass + FemaleAge + FemaleTarsus + MaleAge + MaleTarsus + Treatment*Area + Treatment*TitIndex + Treatment*PFabundance + Treatment*FemaleAge + Treatment*FemaleTarsus + Treatment*MaleTarsus	110 nests
	AdultGT	Treatment + Area + TitIndex + GTeggs + PFabundance + LayingDate + ClutchSize + MeanEggMass + FemaleAge + FemaleTarsus + MaleAge + MaleTarsus + Treatment*TitIndex + Treatment*GTeggs + Treatment*PFabundance + Treatment*FemaleTarsus + Treatment*MaleTarsus	52 nests
	AdultNoGT	Treatment + Area + TitIndex + PFabundance + LayingDate + ClutchSize + MeanEggMass + FemaleAge + FemaleTarsus + MaleAge + MaleTarsus	57 nests
	AdultPF	Treatment + Area + TitIndex+ GTpresence + PFabundance + PFeggs+ LayingDate + ClutchSize + MeanEggMass + FemaleAge + FemaleTarsus + MaleAge + MaleTarsus + Treatment*TitIndex + Treatment*PFabundance + Treatment*PFeggs + Treatment*FemaleTarsus + Treatment*MaleTarsus	98 nests

Tables of parameter estimates of the final models

Table A2. Estimates of treatment differences and other relevant parameters for the overall nest box occupancy analyses of pied flycatchers, based on the final models. In case the final model included an interaction between the treatment effect and a continuous variable we derived pair-wise treatment comparisons for mean, minimum and maximum (with observations in all treatments) values of the continuous variable. Also the treatment-specific main effects for the continuous variable included in the interaction are reported. Estimates with 95% CI excluding zero in bold.

Data set	Variable		Estimate	95% CI
GT sites				
First model	**Treatment x GTeggs interaction**			
	Treatment contrasts when GT eggs = 0:			
		Tit vs. Suitable	0.30	-0.56 – 1.17
		Tit vs. Controls	-0.22	-1.01 – 0.57
		Suitable vs. Controls	-0.53	-1.24 – 0.18
	Treatment contrasts when GT eggs = 2.6 (mean):			
		**Tit vs. Suitable**	**0.79**	**0.07 – 1.51**
		Tit vs. Controls	0.59	-0.04 – 1.21
		Suitable vs. Controls	-0.20	-0.88 – 0.47
	Treatment contrasts when GT eggs = 7:			
		**Tit vs. Suitable**	**1.63**	**0.03 – 3.22**
		**Tit vs. Controls**	**1.98**	**0.56 – 3.39**
		Suitable vs. Controls	0.35	-1.32 – 2.03
	GT eggs effect in different treatments:			
		Tit	0.20	-0.07 – 0.47
		Suitable	0.01	-0.29 – 0.31
		Controls	-0.11	-0.36 – 0.13
	**Area, Oulu**		**-0.91**	**-1.52 – -0.30**
	**PF abundance**		**0.38**	**0.06 – 0.70**
Second model	**Treatment contrasts**			
		**Tit vs. Suitable**	**0.67**	**0.01 – 1.32**
		Tit vs. Controls	0.38	-0.21 – 0.96
		Suitable vs. Controls	-0.29	-0.87 – 0.29
	**Area, Oulu**		**-0.79**	**-1.36 – -0.21**
	**PF abundance**		**0.40**	**0.08 – 0.71**
No GT sites	Treatment contrasts			
		Tit vs. Suitable	0.39	-0.43 – 1.21
		Tit vs. Controls	0.08	-0.43 – 0.60
		Suitable vs. Controls	-0.31	-1.04 – 0.42
	**Area, Oulu**		**-1.95**	**-2.58 – -1.32**
	PF abundance		-0.27	-0.60 – 0.06

Table A3. Estimates of treatment differences and other relevant parameters for the age- and sex-specific nest box occupancy analyses of pied flycatchers, based on the final models. Estimates with 95% CI excluding zero in bold.

Data set	Variable		Estimate	95% CI
Old females	**Treatment contrasts**			
		**Tit vs. Suitable**	**0.72**	**0.07 – 1.37**
		Tit vs. Controls	0.10	-0.42 – 0.62
		**Suitable vs. Controls**	**-0.62**	**-1.20 – -0.04**
	**Area, Oulu**		**-1.68**	**-2.16 – -1.20**
Young females	**Treatment x Area interaction**			
	**Kauhava**			
		**Tit vs. Suitable**	**1.45**	**0.01 – 2.90**
		Tit vs. Controls	-0.15	-1.12 – 0.83
		**Suitable vs. Controls**	**-1.60**	**-2.91 – -0.29**
	**Oulu**			
		Tit vs. Suitable	0.64	-0.24 – 1.53
		**Tit vs. Controls**	**1.02**	**0.18 – 1.86**
		Suitable vs. Controls	0.38	-0.49 – 1.24
Old males	**Treatment contrasts**			
		Tit vs. Suitable	0.65	-0.18 – 1.47
		Tit vs. Controls	-0.15	-0.77 – 0.47
		**Suitable vs. Controls**	**-0.80**	**-1.51 – -0.09**
	**Area, Oulu**		**-1.65**	**-2.29 – -1.00**
	**PF abundance**		**0.41**	**0.06 – 0.76**
Young males	**Treatment contrasts**			
		Tit vs. Suitable	0.63	-0.08 – 1.33
		**Tit vs. Controls**	**0.79**	**0.14 – 1.44**
		Suitable vs. Controls	0.17	-0.52 – 0.85

Table A4. Treatment-specific fitted estimates of timing of breeding, breeding investment and success of pied flycatchers. Estimates are based on the best supported models, except for the egg mass for which the estimates are based on the model including the treatment, study area and female age effects fitted to the full data set (the sites with and without currently breeding great tits combined).

Study area	Treatment	Laying date (1 = 1st May)	Clutch size	Egg mass (g)	Clutch mass (g)	Brood size	Fledgling number
Oulu	Tit	31	6.5	1.68	11.1	6.0	6.0
	Suitable	33	6.5	1.59	11.1	5.9	5.7
	Controls	31	6.5	1.63	11.0	5.9	5.5
Kauhava	Tit	28	6.5	1.60	11.1	6.0	6.0
	Suitable	29	6.5	1.72	11.1	5.9	5.7
	Controls	27	6.5	1.65	11.0	5.9	5.5

Table A5. Estimates of treatment differences and other relevant parameters for the laying date, breeding investment and breeding success analyses, based on the final models. In case the final model included an interaction between the treatment effect and a continuous variable we derived pair-wise treatment comparisons for mean, minimum and maximum (with observations in all treatments) values of the continuous variable. Also the treatment-specific main effects for the continuous variable included in the interaction are reported. Estimates with 95% CI excluding zero in bold.

**Response variable**	**Data set**	**Variable**	**Estimate**	**95% CI**
Laying date	Female	**Treatment contrasts**		
		Tit vs. Suitable	-1.47	-2.99 – 0.04
		Tit vs. Controls	0.53	-0.72 – 1.78
		**Suitable vs. Controls**	**2.01**	**0.64 – 3.37**
		**Area, Oulu**	**3.47**	**2.40 – 4.53**
Clutch size	Female	Treatment contrasts		
		Tit vs. Suitable	0.00	-0.18 – 0.19
		Tit vs. Controls	0.00	-0.15 – 0.15
		Suitable vs. Controls	0.00	-0.17 – 0.17
		Laying date	-0.02	-0.03 – 0.003
Egg mass	Female, GT sites	**Treatment x TitIndex interaction**		
		Treatment contrasts when TitIndex = 0:		
		**Tit vs. Suitable**	**-0.11**	**-0.21 – -0.01**
		Tit vs. Controls	0.02	-0.07 – 0.11
		**Suitable vs. Controls**	**0.13**	**0.03 – 0.22**
		Treatment contrasts when TitIndex = 1.6 (mean):		
		Tit vs. Suitable	0.06	-0.03 – 0.16
		Tit vs. Controls	0.08	-0.01 – 0.16
		Suitable vs. Controls	0.01	-0.06 – 0.09
		Treatment contrasts when TitIndex = 2:		
		Tit vs. Suitable	0.10	-0.01 – 0.22
		Tit vs. Controls	0.09	-0.01 – 0.19
		Suitable vs. Controls	-0.01	-0.10 – 0.07
		TitIndex effect in different treatments:		
		Tit	0.05	-0.01 – 0.11
		**Suitable**	**-0.05**	**-0.10 – -0.01**
		Controls	0.02	-0.003 – 0.04
		Clutch size	-0.03	-0.06 – 0.003
		**Female age, young**	**-0.09**	**-0.15 – -0.03**
	Female, no GT sites	Treatment contrasts		
		Tit vs. Suitable	0.06	-0.04 – 0.15
		Tit vs. Controls	0.00	-0.07 – 0.07
		Suitable vs. Controls	-0.05	-0.14 – 0.04
		Laying date	0.01	-0.001 – 0.01
Clutch mass	Female	Treatment contrasts		
		Tit vs. Suitable	0.01	-0.77 – 0.78
		Tit vs. Controls	0.08	-0.55 – 0.72
		Suitable vs. Controls	0.07	-0.63 – 0.78
		**Female age, young**	**-0.99**	**-1.48 – -0.50**
		**Laying date**	**-0.11**	**-0.18 – -0.03**
Brood size	Adult	Treatment contrasts		
		Tit vs. Suitable	0.02	-0.20 – 0.25
		Tit vs. Controls	0.03	-0.15 – 0.20
		Suitable vs. Controls	0.01	-0.20 – 0.21
		**Clutch size**	**0.15**	**0.07 – 0.23**
Fledgling number	Adult	Treatment contrasts		
		Tit vs. Suitable	0.06	-0.17 – 0.28
		Tit vs. Controls	0.08	-0.10 – 0.26
		Suitable vs. Controls	0.02	-0.19 – 0.23
		**Clutch size**	**0.12**	**0.04 – 0.20**
